# Supplementary material for: Promoting inter-organisational knowledge sharing: A qualitative evaluation of England’s Global Digital Exemplar and Fast Follower Programme
Source: PLoS One. 2021 Aug 2;16(8):e0255220. doi: 10.1371/journal.pone.0255220 (PMC8328305; doi:10.1371/journal.pone.0255220)
Supplement: S1 Appendix — (DOCX) [file pone.0255220.s002.docx]

S1 Appendix: Table 2 GDE/FF pairings, systems, local institutional contexts and mergers

| **Global Digital Exemplar** | **Fast Follower** | **Main Information Technology system** | **In the same**  **regional group?** | **Prior relationship?** | **Other changes** |
| --- | --- | --- | --- | --- | --- |
| Alder Hey (paediatrics)  270 beds | Clatterbridge  (oncology)  103 beds | Meditech | Yes | No |  |
| Cambridge  1,268 beds | University College London Hospital  1000 beds | Epic | No | No |  |
| Sunderland  1010 beds | South Tyneside  N/A | Meditech | Yes | Yes | Merged during GDE |
| Imperial, London  1,412 beds | Chelsea and Westminster  430 beds | Cerner (Imperial)  Meditech (Chelsea and Westminster | Yes | Yes | In partnership |
| Luton and Dunstable  742 beds | Bedford  400 beds | Best-of-Breed / | Yes | Yes | Shared clinicians  Merged April 2020 |
| Newcastle  1,800 beds | Gateshead  600 beds | Cerner (Newcastle)  System C (Gateshead) | Yes | Yes |  |
| Oxford  1,185 beds | Royal Berkshire  687 beds | Cerner | Yes | Yes | Clinicians rotate |
| Royal Free, London  1,770 beds | North Middlesex  443 beds | Cerner  (Royal Free)  System C  (North Middlesex) | Yes | Yes |  |
| Royal Liverpool  850 beds | 1. Liverpool Women’s 2. North Tees and Hartlepool   572 beds | Best of Breed  Best of Breed based on TrakCare | 1. Liverpool Women’s – Yes 2. North Tees and Hartlepool- No | Liverpool Women’s – yes  North Tees and Hartlepool - no | Liverpool Women’s – shared CIO and executive team with Royal Liverpool and merger into Liverpool Hospitals Trust |
| Salford Royal  828 beds | Pennine Acute  N/A | Allscripts | Yes | Yes | Merged 2017 |
| Taunton and Somerset  660 beds | Wye Valley  320 beds | Best of Breed based on  IMS-Maxims | No | Yes – based on same system | Taunton merged with local mental health Trust 2020  Wye Valley merged with Gloucestershire 2020 |
| Birmingham  2,366 beds | Heart of England  N/A | Best of Breed – in -house | Yes | Yes | Merged pre-GDE Programme |
| Bristol  1085 beds | Whittington  346 beds | System C | No | No |  |
| Southampton  1,100 beds | Hampshire  806 beds | Best of Breed | Yes | No | Merged procurement team as a result of GDE Programme |
| West Suffolk  442 beds | Milton Keynes  457 beds | Cerner | No | No |  |
| Wirral  855 beds | Countess of Chester  625 beds | Cerner | Yes | Yes | Shared departments and clinicians |
| **Mental Health Trusts** |  |  |  |  |  |
| Worcestershire  403 beds | Sheffield  72 beds | RIO and Best of Breed (Worcester)  Self-build (Sheffield) | No | Yes |  |
| Berkshire  323 beds | Lancashire  518 beds | RIO | No | No |  |
| Birmingham and Solihull  702 beds | Coventry and Warwickshire  N/A | RIO | Yes | Yes |  |
| Oxford Health  562 beds | Sussex Partnership  588 beds | Advanced | No | No |  |
| Mersey Care  766 beds | North West Boroughs  297 beds | RIO | Yes | Yes | Previously implemented RIO together |
| Northumberland Tyne and Wear  N/A | Cumbria Partnership | RIO | Yes | Yes | Merged 2020 |
| South London and Maudsley  786 beds | Oxleas  N/A  South West  London and St Georges  391 beds | RIO | Yes | Yes |  |
