# Supplementary material for: Promoting inter-organisational knowledge sharing: A qualitative evaluation of England’s Global Digital Exemplar and Fast Follower Programme
Source: PLoS One. 2021 Aug 2;16(8):e0255220. doi: 10.1371/journal.pone.0255220 (PMC8328305; doi:10.1371/journal.pone.0255220)
Supplement: Attachment — Submitted filename: Response to Reviewers.doc [file pone.0255220.s003.doc]

**Reviewer 1**

**Point 1: The abstract indicates that the research draws on socio-technical systems theory. The introduction might explain what this is and how it orients the research in a particular way.**

Response: We have expanded the Introduction and inserted a citation to explain that we draw on perspectives surrounding sociotechnical systems, which assume that technological and social factors are interrelated and shape each other in complex ways.

**Point 2: Figure 1 comes at the end of the discussion. Something should be said about the figure in the text, i.e. it is a summary ….**

Response: We have moved mention of the Figure to the beginning of the Results section as it is intended to provide an overview of factors promoting informal networking between Global Digital Exemplar and Fast Follower sites. We also now describe the Figure in the text.

**Point 3: Line 360 where references are made to the literature – should the text read something like ‘networking, consistent with findings in other contexts’ since the literature is not specifically about health systems. The subtitle indicates it is a discussion of the wider literature but it would help to clarify here.**

Response: Thank you. We have, as suggested, inserted “…consistent with findings in other contexts”.

**Point 4: Line 372 should the sentence starting ‘This is illustrated …’ read something like. These findings from studies in the socio-technical systems tradition are illustrated by GDEs and FFS in our sample that were sharing knowledge …**

Response: Thank you for this suggestion. We have revised the sentence to read: “These findings from studies in the sociotechnical systems tradition are illustrated by GDEs and FFS in our sample that were sharing knowledge with other provider organisations outwith their formal pairings.”

**Point 5: Line 385 ‘These strategies partnerships …’ Here is this in reference to your empirical study of partnerships or is this statement made in relation to similar partnerships. Unclear whether the discussion in this section is meant to refer to this study primarily or to generalisations from it to similar situations.**

Response: We have made clearer throughout the paragraph that the discussion is based on our findings. In doing so, we have revised the first sentence to read: “The strategic partnerships observed in our work were formed through various mechanisms and channels of communication, both formal and informal. Knowledge sharing is therefore difficult to plan and may have many unanticipated benefits and/or difficulties.”.

**Point 6: Line 279. Should it read ‘the GDE site meant a clinician [came or could come] over and could test their system’**

Response: Apologies for this typo. We have inserted “could” as suggested.

**Point 7: Line 308 greater likelihood [of] priori linkages**

Response: Apologies, now corrected.

**Point 8: Reference 29. Citation seems incomplete.**

Response: This has now been corrected.

**Reviewer 2**

**Point 1: What is the digital maturity of the participants? The complexity and sophistication of the knowledge transfer regarding implementing an EMR is quite different to sharing knowledge for implementing a basic LIS.**

**It was difficult to truly understand the program from the text. Would it be possible to represent the program diagrammatically? Map out the relationships of the GDEs and FFSs and perhaps indicate the size and digital maturity of each facility in the diagram and the nature of the relationships. How many were positive, negative, neutral? Which ones had unidirectional or bidirectional information flows? This would allow a more detailed and granular understanding of the program, particularly for international readers.**

Response: Thanks for this interesting suggestion. We have added extra detail when describing the sample in the Methods section to make clear that all sites participating in the Programme had relatively high levels of digital maturity, and in most cases embarked on major upgrades in core information infrastructures such as Electronic Health Record (EHR) implementation/optimisation as part of the Programme. We have also included a diagram illustrating dynamics of how the knowledge transfer between GDEs and FFs was intended to take place. This paper focuses on exploring the processes surrounding knowledge transfer and we examine factors seen as affecting this. However, there is not scope in this short paper to analyse the influence of size and maturity on the relationship and outcomes of each GDE/FF pairing. Such a discussion would inevitably allow identification of individual sites and thus compromise our commitments to maintain anonymity of provider organisations participating in the evaluation. We are drafting a further paper on the impact of GDE in transforming participating organisations that discusses (albeit also briefly) the impact of size and maturity/prior momentum.

**Point 2: Did digital maturity across the NHS as a whole increase during the GDE program? I take the author's point that it is impossible to attribute any granular outcomes to the program, however I think it is important to understand did the NHS as a whole complex adaptive system, continue to undertake digital transformation? How many EMRs were implemented during the GDE program? How many other applications were implemented? It would be critical to know, for example, if the number of EMRS deployed actually decreased or stalled during the program. Conversely, it would strengthen the conclusion and discussion of the paper to know how many systems were implemented in the FFS during the program. There is no data to suggest how many applications were actually adopted by the FFs during the program, which as I understand it, was the primary intent of the program. IF interorganisational knowledge transfer was effective, we would at least expect ongoing implementation of new systems in the FFs. and this should be reported.**

Response: Table 3 indicates the core EHR/EMR provider for each provider participating in the programme. We do not have data that would allow us to compare the GDE participants with the NHS as a whole. There was an array of other apps in each site which cannot be readily summarised. We are preparing a further paper that will analyse the evolving EHR etc market. This paper focuses on the overall success of the Programme in stimulating digitally enabled transformation and the sharing of knowledge between participating provider organisations. GDE/FF partnerships were one knowledge sharing mechanism that helped, together with others e.g. Blueprinting, to promote an ethos of learning to promote Programme aims. We have revised the Discussion section to indicate this more clearly.

**Point 3: Be consistent on the version of NVivo used, it changes throughout text**

Response: Apologies – this has now been corrected to read Version 11 throughout.

**Reviewer 3**

**Point 1:** **Abstract**

**The results state that partnerships enhanced learning and accelerated adoption. However, the study does not attempt to answer this question. It assesses users' opinions of the pairings, and there is no comparison to non-paired arrangements. The conclusions and language in the actual paper is more moderate and I think the abstract should reflect this.**

Response: Thank you. We have revised the Abstract to reflect that the findings were based on user perceptions and not on quantitative measures of learning and adoption.

**Point 2:** **Methods**

**The study deliberately sampled members of local GDE teams via the GDE program manager. Presumably many (if not most) of these individuals' jobs are funded by- and dependent on the success of this program. How was this potential conflict of interest and bias in sampling managed?**

Response: Reviewer 3 is correct in that many of the sampled participants may have been biased towards successful accounts of the Programme, although we attempted to compensate for this by deliberately snowball sampling additional participants with different perspectives. We now make this clearer in the Methods section and also acknowledge this as a limitation in the Discussion.

**Point 3: Data collection (Table 2). The table outlines the type of data collection methods. However, we are not shown the proportion of data collected (interviews, meetings, etc.) from GDE v FF. Given that there were twice as many in depth case study sites that were GDEs (p5 Line 96), is this sample disproportionately from a GDE perspective? Again a perspective that might be more likely to be positive.**

Response: We have revised Table 2 to show the type of data collected in GDEs and FFs and have included the limited data collected in FFs as a potential limitation of the study in the Discussion section.
