# Supplementary material for: Promoting inter-organisational knowledge sharing: A qualitative evaluation of England’s Global Digital Exemplar and Fast Follower Programme
Source: PLoS One. 2021 Aug 2;16(8):e0255220. doi: 10.1371/journal.pone.0255220 (PMC8328305; doi:10.1371/journal.pone.0255220)
Supplement: Attachment — Submitted filename: Response to reviewers.doc [file pone.0255220.s004.doc]

**Response to Reviewers**

**Reviewer 3 
Point 1: With respect to the comment about presenting qualitative findings as quantitative conclusions (especially in the abstract),**^[1]^ **this has been addressed in the results summary in the abstract but the conclusion. This type of language still exists throughout the main manuscript, even though the limitations section has been expanded.**

Response: We have made clear in the Results and Discussion sections that the findings were based on user perceptions and not on quantitative measures of learning and adoption.

**Point 2: With respect to the snowballing and targeting negative perceptions in the methods, how was this done? How would a researcher at another centre reproduce the method to gain comparable results?**

Response: We asked interviewees for recommendations of further participants to interview, asking explicitly for those with differing involvements and varied viewpoints on the GDE Programme. We have now made this clearer in the Methods section.

**Point 3: Figure 1 is a simplified schematic that in my opinion doesn't address the suggestion from reviewer 2 which I think would add a great deal to the paper.**^[2]^ **This aligns with reviewer 3 comment 3. It doesn't have to be a detailed analysis of the influence of size and maturity on the results, just a representation of these aspects and relationships of the study participants to better contextualise the findings.**

Response: We have amended Figure 1 to represent the flow of knowledge from GDEs to FFs as intended in the GDE Programme and we now describe in more detail in the text how practice varied from this plan. We have, in addition, substantially reworked the detailed breakdown of GDE/FF pairings in Table 2 (Appendix). We have added a column noting any prior relationships between GDE and FF and indicate the size of the hospitals by including numbers of beds (where available). We have replaced specific details of local regional groupings with a Yes/No column on whether GDE and FF are in the same regional group.

In almost all of the GDE and FF pairings the knowledge transfer was bi-directional. We cannot add this information relating to specific providers as this would compromise the agreed terms of access for the evaluation which included a firm commitment not to identify specific provider organisations or individuals.

Finally, assessments of the digital maturity of providers across the NHS (based on self-assessments) are held centrally within the NHS and not publicly available. We did not collect data on the number of electronic health records and other digital systems implemented in the rest of the NHS during the time period of the GDE Programme. We are therefore unable to add this context to the paper and have noted this in the Discussion section as a limitation.

We trust that these revisions are to your satisfaction and that we are now in a position to proceed with publication. Please do not however hesitate to contact me if you require any further details.

With kind regards,

Susan Hinder (on behalf of the co-authors)

1. Reviewer 3 in previous response: The results state that partnerships enhanced learning and accelerated adoption. However, the study does not attempt to answer this question. It assesses users' opinions of the pairings, and there is no comparison to non-paired arrangements. The conclusions and language in the actual paper is more moderate and I think the abstract should reflect this.
2. Reviewer 2 in previous response:

   What is the digital maturity of the participants? The complexity and sophistication of the knowledge transfer regarding implementing an EMR is quite different to sharing knowledge for implementing a basic LIS.

   It was difficult to truly understand the program from the text. Would it be possible to represent the program diagrammatically? Map out the relationships of the GDEs and FFSs and perhaps indicate the size and digital maturity of each facility in the diagram and the nature of the relationships. How many were positive, negative, neutral? Which ones had unidirectional or bidirectional information flows? This would allow a more detailed and granular understanding of the program, particularly for international readers.

   Did digital maturity across the NHS as a whole increase during the GDE program? I take the author's point that it is impossible to attribute any granular outcomes to the program, however I think it is important to understand did the NHS as a whole complex adaptive system, continue to undertake digital transformation? How many EMRs were implemented during the GDE program? How many other applications were implemented? It would be critical to know, for example, if the number of EMRS deployed actually decreased or stalled during the program. Conversely, it would strengthen the conclusion and discussion of the paper to know how many systems were implemented in the FFS during the program. There is no data to suggest how many applications were actually adopted by the FFs during the program, which as I understand it, was the primary intent of the program. IF inter-organisational knowledge transfer was effective, we would at least expect ongoing implementation of new systems in the FFs. and this should be reported.
